# Female Genitalia Concealment Promotes Intimate Male Courtship in a Water Strider

**DOI:** 10.1371/journal.pone.0005793

**Published:** 2009-06-10

**Authors:** Chang S. Han, Piotr G. Jablonski

**Affiliations:** 1 Laboratory of Behavioral Ecology and Evolution, School of Biological Sciences, Seoul National University, Seoul, South Korea; 2 Center for Ecological Research, Polish Academy of Sciences, Dziekanow Lesny, Lomianki, Poland; Queens University, Canada

## Abstract

Violent coercive mating initiation is typical for animals with sexual conflict over mating. In these species, the coevolutionary arms-race between female defenses against coercive mating and male counter-adaptations for increased mating success leads to coevolutionary chases of male and female traits that influence the mating. It has been controversial whether one of the sexes can evolve traits that allow them to “win” this arms race. Here, we use morphological analysis (traditional and scanning electron micrographs), laboratory experiments and comparative methods to show how females of a species characterized by typical coercive mating initiation appear to “win” a particular stage of the sexual conflict by evolving morphology to hide their genitalia from direct, forceful access by males. In an apparent response to the female morphological adaptation, males of this species added to their typically violent coercive mounting of the female new post-mounting, pre-copulatory courtship signals produced by tapping the water's surface with the mid-legs. These courtship signals are intimate in the sense that they are aimed at the female, on whom the male is already mounted. Females respond to the signals by exposing their hidden genitalia for copulatory intromission. Our results indicate that the apparent victory of coevolutionary arms race by one sex in terms of morphology may trigger evolution of a behavioral phenotype in the opposite sex.

## Introduction

Evolutionary conflict between the sexes may lead to direct coercive mating (sensu [1]) in which a male forcefully opens the female genitalia and inserts his genitalia in order to transfer sperm, regardless of female compliance. Although Eberhard [1] argued that direct coercion may be rare in insects due to the lack of sufficient genitalic force required to open the female genitalia, some insect taxa are regarded as good examples of the direct coercive mating system.

Water striders, Gerridae, are a classical example of sexually antagonistic selection (sexual conflict) that produces the direct coercive mating system, in which males forcefully initiate intromission right after mounting a female [2]. Typical mating behavior of many species of the genus *Gerris* and *Aquarius* can be described as follows: a male mounts a female without any apparent courtship behavior, grasps the female's thorax, overcomes female resistance, and then inserts his genitalia into the female genitalia through the vulvar opening between the gonocoxae [2], [3]. Research on water striders revealed that the sexual-conflict driven coevolutionary arms race between female defenses against mating and male counter-adaptations for increased mating frequency gave rise to coevolutionary chases ([4]; cycling of arms level between sexes) of male and female external morphologies which influence male mating success. Among the morphological traits studied in this context, morphology affecting the degree of concealment of the female genitalia has not been considered.

**Figure 1:**
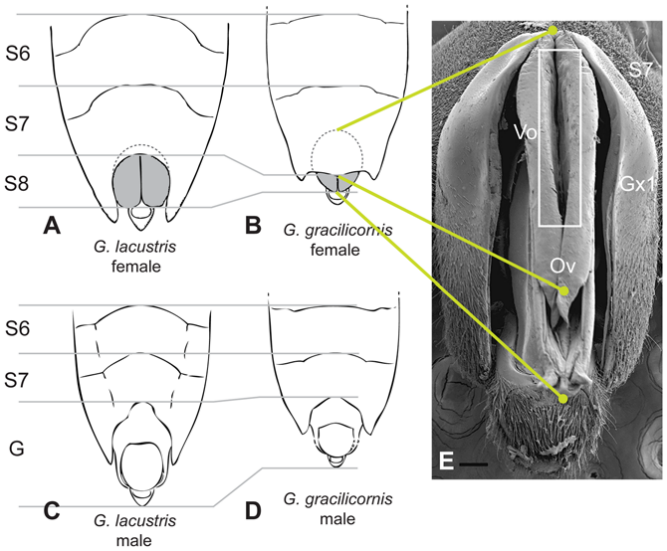
Genital segments in a typical Gerris and in *G. gracilicornis.* Schematic drawings of ventral views of abdominal tips of (A) female *G. lacustris*, (B) female *G. gracilicornis*, (C) male *G. lacustris* and (D) male *G. gracilicornis*. Drawing (B) corresponds to (E) SEM image of the posterior view of a partially inflated female genital segment with gonocoxae 1 spread apart and the ovipositor tube visible. G - genitalia; Gx1 - gonocoxa 1; S6 - sixth segment; S7 - seventh segment; Ov - ovipositor; Vo - vulvar opening. Scale bar: 0.1 mm. (A)-(D) were modified from Andersen [13]. The broken lines schematically indicate the difference between a typical Gerridae and *G. gracilicornis* in the portion of S8 that is hidden within S7.

The shape of the posterior margin of a typical *Gerris* female's pre-genital part is concave [5], and it does not cover the base of the female genitalia (Figure 1A). Therefore, female genitalia are well exposed and the vulvar opening appears to be easily available to forcefully mating males [2]. It may be assumed that if the female genitalia were less exposed, then direct coercion would not be possible (Figure 1B). An overview of figures found in the literature [6] and direct examination of live specimens drew our attention to an apparently unique water strider species, *Gerris gracilicornis*. Due to the morphology of their pre-genital segment, *G. gracilicornis* females appear to have their genitalia relatively well hidden in comparison to the females of other species (Figure 1 ABCD). Hence, it is feasible to hypothesize that the vulvar opening, through which the male genitalia enter during intromission, is well shielded behind the pre-genital segment.

The aim of this research was to investigate the anatomy of female genitalia in *G. gracilicornis*, to study the evolutionary history of the external morphology of female genitalia (concealment of segment 8 behind segment 7) in water striders, and to discuss its effects on male mating behavior in *G. gracilicornis* – a species with the most extremely concealed female genitalia among the species of *Gerris, Aquarius* and *Limnoporus*. Studies on the evolution of genitalia in internally fertilizing animals have mostly focused on coevolution between the internal morphology of female genitalia and the external morphology of male genitalia (e.g. [7], [8]). In this report, we show that the morphology-dependent concealment of female genitalia in *G. gracilicornis* might have triggered the evolution of male behavioral traits, such as the post-mounting, pre-copulatory courtship signals produced as ripple waves on the water's surface.

## Results

### Morphology of female external genitalia

**Figure 2:**
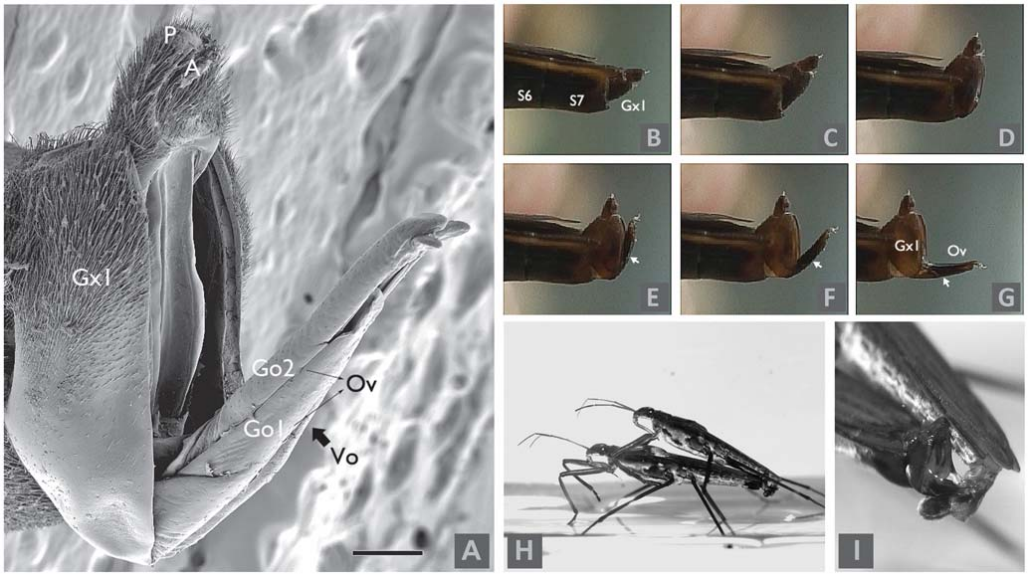
Role of female genitalia anatomy in mating interactions of *G. gracilicornis*. (A) Scanning electron micrographs of partially inflated genital segment of female *Gerris gracilicornis*. A, anal opening; Gx1, gonocoxa 1; Go1, gonapophysis 1; Go2, gonapophysis 2; Ov, ovipositor; P, proctiger; Vo, vulvar opening. Scale bar: 0.2 mm. (B)-(G) Sequence of genitalia inflation of female *Gerris gracilicornis* in a lateral view. (B) un-inflated gonocoxae 1 in segment 7. Gx1, gonocoxa 1; S6, segment 6; S7, segment 7. (C)∼(F) represent spreading out of gonocoxae 1 with protruding of the ovipositor (gonapophyses). Arrow head indicates the vulvar opening. (G) represents fully inflated female genitalia with protruded ovipositor (Ov). In B-G the body was squeezed to imitate the protrusion observed during mating interactions. (H): Copulating pair of *Gerris gracilicornis*. (I): Interdigitation of male and female genitalia. The phallus is inserted and clasped between the female's gonapophyses. The phallus enters the oviposition tube through the vulvar opening between the two gonapophyses 1.

Generally, the external components of *G. gracilicornis* female genitalia are similar to those in other species of *Gerris*. Segments 8 and 9 form two pairs of gonocoxae (Figure 1E and 2A). Exposed segment 8 is divided along the ventral line into the cylinder shaped first gonocoxae, which carries the first gonapophyses and covers other genital segments. Segment 9 forms the second gonocoxae, carrying the second gonapophyses. The first gonapophyses from the first gonocoxae are placed below the second gonapophyses from the second gonocoxae. These two pairs, first gonapophyses and second gonapophyses, form an ovipositor [9]–[11]. The ovipositor is concealed in the first gonocoxae. The vulvar opening is placed in the gap between the two pairs of gonapophyses (Figure 1E and 2A). As Fairbairn et al. [2] observed in *Aquarius remigis*, the vulvar opening in *G. gracilicornis* is also located approximately in the middle (about 50%) of the length of the ovipositor, not in the distal end of the ovipositor (Figure 1E and 2A). Therefore, water strider males should not be able to achieve coercive intromission when 50% of the female gonocoxae is concealed. Hence, it appears that in *G. gracilicornis*, where 77.7% (3.3%; *N* = 9) of the length of the female gonocoxae is concealed (Figure 1 and S2), males are not able to insert their genitalia coercively.

### Description of mating interactions leading to copulation

We watched 25 mating interactions, the majority of which progressed in the following manner. A male forcefully mounts a female and presses the tip of his abdomen (genitalia) against the end of hers. After a few minutes of courtship signals (see description below), the female's gonocoxae, concealed in segment 7, protrudes perpendicular to the body axis, and the anal opening is exposed (First protrusion, Figure 2BCD). At this time, the male phallus cannot enter the gonocoxae, because they are not yet fully open. After a few seconds or minutes, the gonocoxae open and the ovipositor is lowered (Figure 2E). The ovipositor is then protruded parallel to the body axis (Full protrusion, Figure 2FG). Finally, the inflated male phallus wraps the ovipositor, clasps the gonapophyses, and enters the vulvar opening (Figure 2 HI). It is clear that if the female genitalia remain hidden (Figure 2BC) males *G. gracilicornis* can not achieve successful intromission. Hence, it is the female who decides when, after an apparently coercive mounting and genitalia attachment, copulation (intromission) actually begins, or whether it takes place at all. Male signaling ends after intromission is achieved.

**Figure 3:**
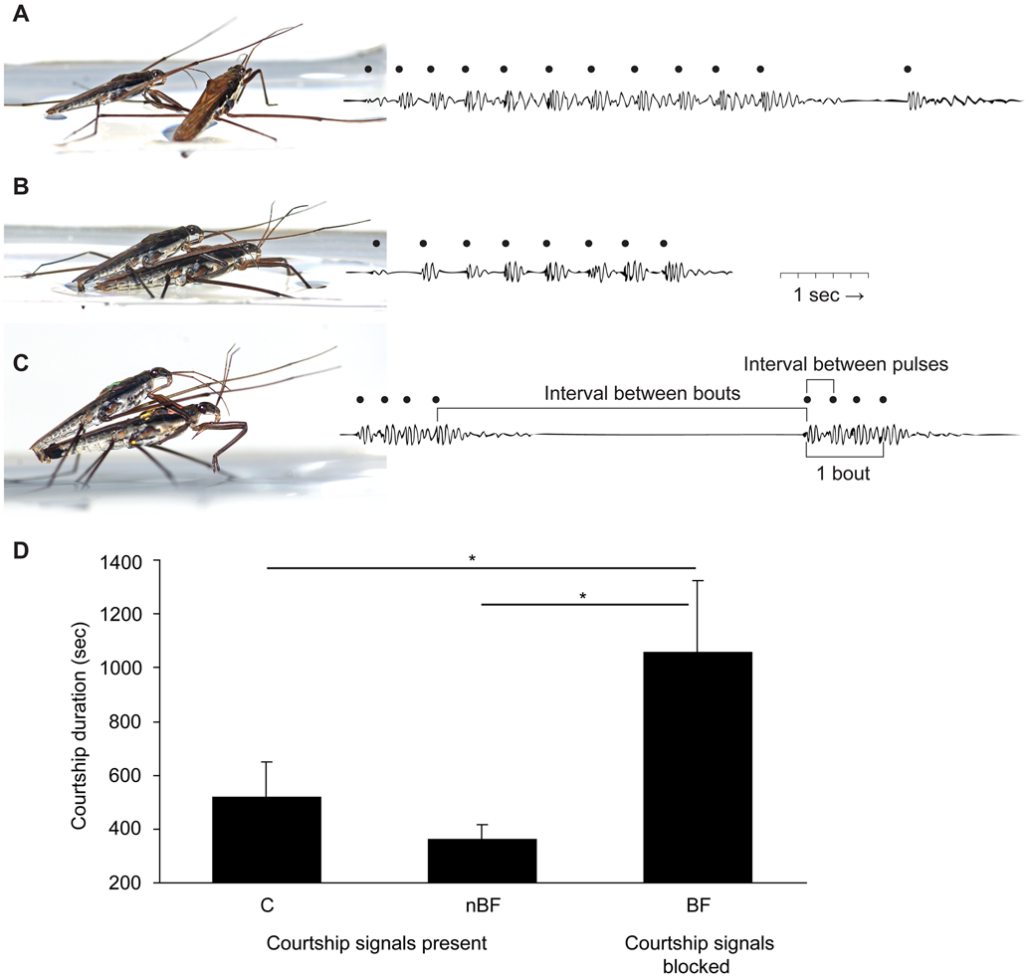
Male signaling and its function in *G. gracilicornis*. Examples of (A) Grasping signals, (B) Mounting signals, and (C) Attachment signals. Black dots above the waveform displays of ripple signals indicate the moment the mounting male's mid-legs hit the water surface. The ripple wave is produced on the water's surface by male mid-legs as well as by movements of male and female bodies induced by male mid-leg movement. (D) Effect of male pre-copulatory signals on courtship duration; C – control treatment (22 unmanipulated females; male signals present); nBF – “non-effective bar” treatment (21 females with a bar attached along her thorax; male signals present); BF – “w-bar” treatment (19 females with a w-bar attached across thorax for blocking male signals). Means andSE are shown. * *p*<0.05.

Moreover, in 25 mating interactions, we also measured the timing of signaling and intromission, and described the detailed behavioral context of the signaling. *G. gracilicornis* males produce ripple signals with their mid-legs stretched forward, vibrating vertically (Figure 3 and Video S1). These signals are produced from the time a male grasps or mounts the female until full intromission is accomplished (after the female fully protrudes her ovipositor; Videos S2 and S3). After complete intromission, males discontinue signaling (Video S3). Thus, the correlation between the time of full ovipositor protrusion and the time signaling ceased was 1 (*R^2^* = 1.00, *N* = 25). If the ovipositor is not fully protruded, the male resumes signaling. Since it takes a few seconds or minutes to go from partial horizontal protrusion (like in Figure 2CD) to full ovipositor protrusion (like in Figure 2 EFG), mounting males repeatedly produce signal bouts (Video S3). Finally, vertical protrusion of the gonocoxae was observed, followed by full protrusion of the female ovipositor and successful intromission of the male phallus. This behavior indicates that the male's signals can be viewed as a means of courtship to induce female acceptance of intromission.

### Signals and their function

The basic unit of all signaling consisted of a single tap on the water's surface with the mid-legs (we call it a pulse, because it created a short pulse of ripples on the water's surface, Figure 3ABC; Video S1). The pulses were produced in bouts. A bout consisted of several taps on the water's surface, followed by an interval, during which the male did not produce any pulses. Three types of signaling behavior have been identified for *G. gracilicornis* based on their context and temporal patterns: 1) grasping signals, 2) mounting signals, and 3) attachment signals. “Grasping signals” are produced from the time a male grasps the female until the time the male aligns his body parallel to the female's body and grasps her midcoxa with his forelegs (Figure 3A). Males produce irregular numbers of *pulses* per *bout* with their mid-legs stretched wide (Table S1 and Video S1). “Mounting signals” are produced from the time the male properly mounts the female to the time the male genitalia successfully attaches to the surface of a female gonocoxae (Figure 3B; before intromission of male genitalia is achieved). At this stage, the male produces *pulses* at irregular intervals, with mid-legs stretched forward, parallel to each other (Video S1). Finally, “attachment signals” are produced from the moment of genitalia attachment till the moment the female protrudes her ovipositor and the male's genitalia get hold of it (Figure 3C), at which point intromission occurs. At this final stage, relatively regular numbers of *pulses* are produced per *bout*, with mid-legs stretching parallel to each other (Videos S1 and S2). Additionally, antennal drumming of the female body by males also occurred; male antenna bent downward and tapped the female head and antenna according to the same pattern as the simultaneously produced ripple signals. More statistical details regarding the signals are displayed in Table S1 and the accompanying text, which compares them with other ripple signals known in Gerridae.

To test the hypothesis that male signals induce females to fully protrude their genitalia we observed female mating behavior after blocking the attachment signals. We predicted that in this experimental situation the time until full protrusion of the female's ovipositor would be longer than in the case of the control, or that genitalia protrusion would not occur at all. By attaching a w-shaped metal bar (see Figure S1 and methods for details) on the top of the female thorax, the number and intensity of male attachment signals was severely reduced, but not totally extinguished. The protrusion of female gonocoxae was delayed significantly with the w-bar (BF) treatment in comparison to the control (C) treatment (Figure 3D, C-BF, Wilcoxon matched pairs test, *Z* = 2.40, *N* = 21, *p* = 0.016, *p_B_* = 0.047). This delay in protrusion was not caused by a mere addition of weight to the female, since there was no difference in the latency of protrusion between the control and nBF treatments which consisted of a bar attached along the female's horizontal axis (Figure 3D, C-nBF, Wilcoxon matched pairs test, *Z* = 0.32, *N* = 20, *p* = 0.748, *p_B_* = 0.748). There was a difference, however, between the BF and nBF treatments (Figure 3D, nBF-BF, Wilcoxon matched pairs test, *Z* = 2.33, *N* = 19, *p* = 0.02, *p_B_* = 0.04) since male ripple signals were still effective in spite of loading. Generally, genitalia inflation of *G. gracilicornis* females was achieved within 15 minutes of mounting. However, courtship duration for pairs with bar-attached females (BF treatment) was over 15 minutes in only 47.6% of mating pairs (10 out of 21 pairs).

### Comparative analysis

Male mating behavior was divided into three categories (see methods for details):

(1) simple direct coercive mating behavior typical for most Gerridae (DC; 13 species), where a male forcefully inserts his genitalia into the female's;

(2) direct coercive mating mixed with an alternative tactic of pre-mounting courtship signaling to “persuade” (sensu [1]) females to mate (DC/P1; 3 species);

(3) intimate post-mounting courtship signaling to persuade the female to protrude her genitalia for intromission, after coercive mounting - characteristic for *G. gracilicornis* (P2; this study).

**Figure 4:**
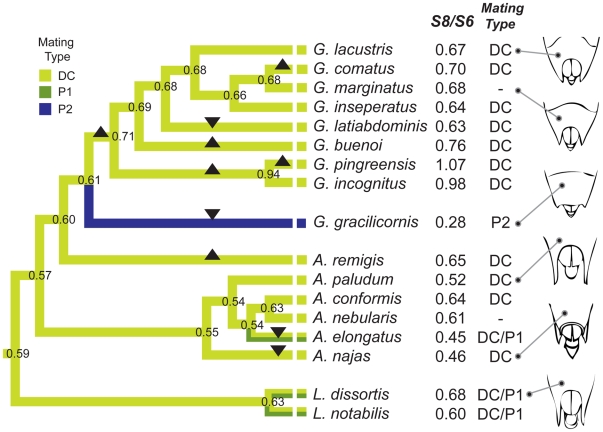
Hypothetical phylogenetic history of mating types and S8/S6 ratio in Gerridae. Changes of the color of branches represent the evolution of mating types. Squares at the tips of the terminal branches indicate current mating types. Numbers beside the terminal branches are mean S8/S6 ratios of species (see Table S2). Ancestral S8/S6 ratios at each node were produced by PGLS method using COMPARE 4.6b [39]. Triangles (pointing up) indicate significant increases in S8/S6 ratio during evolution. Inverted triangles (triangles pointing down) indicate significant decreases in S8/S6 ratio. Pictures of female abdominal tips were modified from Andersen [13], Andersen & Spence [51] and Stichel [52]. DC, direct coercive mating; P1, Persuasive mating type 1; P2, Persuasive mating type 2.

Ancestral reconstruction showed that the DC/P1 tactic evolved from direct coercion (DC) at least twice (Figure 4), while the P2 tactic evolved once - in our study subject, *G. gracilicornis*.

We used the ratio of the length of the visible genital segment 8 to segment 6 (S8/S6; Figure 1) as an approximate index of the proportion of the genital segment length that is not shielded within segment 7 (see Methods and Figure S2 for validation of this method in three species of Gerridae). The smaller is the ratio S8/S6, and the larger is the degree of concealment of the female genitalia. Ancestral reconstruction of the S8/S6 ratio indicates that, among ten significant evolutionary changes, trends toward a decreasing degree of exposure of the female genitalia (S8/S6) are present in four lineages: *A. elongatus* (*Z* = −3.53, *p*<0.0001), *A. najas* (*Z* = −4.41, *p*<0.0001), *G. gracilicornis* (*Z* = −16.17, p<0.0001) and *G. latiabdominis* (*Z* = −2.45, *p* = 0.0142). Two of these evolutionary changes occurred within the “direct coercion” (DC) mating system, one was associated with a switch from DC to DC/P1 (*A. elongatus*), and one occurred in the lineage of *G. gracilicornis* (P2), where the level of decrease in the S8/S6 ratio was exceptionally noticeable. The S8/S6 ratio in *G. gracilicornis* (S8/S6 = 0.28) is lower than in any other Gerridae (Figure 4 and Table S2), indicating the highest degree of female genitalia concealment among water striders. This is due to a concave segment 7 (Figure 1), which represents the highest ratio of segment 7 to segment 6 (S7/S6; a relative index of the size of S7; Table S2) among *Gerris sp.*, with a value of 1.30.

Two evolutionary changes in *A. najas* and in *A. elongatus* produced a relatively extensive concealment of the female genitalia, with only about 45–50% (S8/S6 values are 0.45 and 0.46) of the female genitalia (segment 8) exposed. This indicates that the distal edge of segment 7 may be located roughly over the region of the vulvar opening, through which coercive intromission is performed.

## Discussion

### Do G. gracilicornis females “win” the evolutionary race over initiation of copulation?

Following cautionary notes by Arnqvist & Rowe [12], we use the phrases, “females gained advantage in the sexual conflict over mating initiation” or “females won the coevolutionary race over mating initiation” in the narrow-sense, indicating that mating initiation is largely determined by females. Hence, “winning” or “gaining advantage” does not imply a fitness advantage, but merely winning a particular stage of behavioral control over mating initiation.

Water striders are characterized by a classic direct coercive mating system shaped by the evolutionary sexual conflict between males, attempting to mate more frequently than the female's evolutionary interest would dictate [3], [12], and females, opposing these mating attempts. Based on our results, we propose that the typical lack of concealment of female genitalia in Gerridae, contributes to the existence of this coercive mating system and the antagonistic coevolution between males and females. The female genital segments of most Gerridae species are only partially concealed in the pre-genital segment (abdomen segment 7; [13]), and the gonocoxae are sufficiently exposed for a male to forcefully insert his phallus into the vulvar opening, as described in *A. remigis* [2], where S8/S6 = 0.65. Females in some Gerridae evolved antagonistic adaptive external morphologies such as abdominal spines in the last abdominal segment [14], [15] and downward-bent abdomens [16], [17], or defensive behaviors like jumping, and rubbing to fight off the male mating attempts [3]. In response to the females' adaptations males evolved counter-adaptations, such as downward-bent abdomens and genital morphology [14], [16], [17] which increases the efficiency of forceful mating initiation [18].

According to published data, there appears to be no consistent trend in favor of any of the two sexes winning this evolutionary conflict over mating initiation [16], [17]. However, our results have shown that the large convex pre-genital segment 7 of *G. gracilicornis* females evolved to cover 70–80% of the gonocoxae length (Figure 1E). Because the vulvar opening, where the male phallus is inserted during copulation, is located at about half the ovipositor length, we observed that *G. gracilicornis* males, unlike males of the remaining Gerridae, cannot forcefully insert their phalli into the vulvar opening without the female's acceptance. Therefore, unlike males of the remaining Gerridae, *G. gracilicornis* males are unable to commence copulation even after coercively overcoming female resistance to mounting, and despite positioning their genitalia against the female's abdominal tip (the gonocoxae). We propose that the morphological evolution of segment 7 in *G. gracilicornis* may be viewed as an example of females gaining full advantage over males in the evolutionary conflict over mating initiation, at least as far as the initiation of intromission is concerned. Hence, we propose that, similar to the evolution of female abdominal spines and other external morphologies in some water striders [14]–[17], female genitalia concealment in *G. gracilicornis* might have evolved as an effective mating-resistance trait under sexual-conflict driven selection. Four significant changes towards genitalia concealment among species practicing direct coercive mating seem to fit this idea. However, four other significant evolutionary changes towards more extensive exposure of female genitalia (see Figure 4) indicate that other selective factors may also affect this trait. We are not aware of selection mechanisms that may favor, in the context of coercive mating initiation, more exposed female genitalia in water striders. We suspect that one of the advantages of more exposed female genitalia may be related to species-specific egg laying behavior. For example, if females need to reach underneath floating leaves to oviposit there, longer exposed ovipositor and narrower segment 7 with an indent in the middle (shapes presented in Figure 4) may be favored. Future comparative analyses of detailed descriptions of egg laying and female genitalia morphology may determine plausibility of this hypothesis. Hiding genitalia behind a shield of segment 7 is only one of many ways to achieve control over mating initiation by female insects. In general, regardless of the genitalia exposure, female insects who are able to regulate the opening of gonocoxae or vulvar opening appear to control intromission (see [18]). However, based on detailed descriptions of mating in water striders with exposed female genitalia [2] and given that males of these species do not produce courtship signals, it seems that females in those species cannot control copulation initiation. In these species, coevolutionary arms race between sexes may be shifted to the post-intromission period. For example, females may internally control the sperm location or fertilization by specific males [19]. We cannot exclude the possibility that male signals observed in some of these water strider species at the later stages of mating interactions (e.g. [20]–[23]) might have evolved in the context of such female internal adaptations.

### Evolution of post-mounting courtship signals and female genitalia concealment

We described previously unknown pre-copulatory courtship signals, produced by *G. gracilicornis* males during coercive mating initiation attempts (grasping and mounting signals), and after direct coercive mounting of females (attachment signals). We demonstrated that the function of the attachment signals is to induce protrusion of otherwise hidden female genitalia for the initiation of copulation (intromission).

The post-mounting attachment signals of *G. gracilicornis* males might have evolved from some of the behaviors observed during pre-copulatory interactions in most Gerridae: short signals given while attempting to mount females or leg movements observed after mounting in the initial phase of mating. The former, short “grasping and mounting signals,” were observed by us in *G. latiabdominis* and *A. paludum* (Han, personal observation), and we suspect that they may exist in many Gerridae, but have simply eluded the attention of most researchers until now. We hypothesize that the evolutionary transition from these behaviors (“grasping and mounting signals”) towards “post-mounting courtship signals,” represented by attachment signals in *G. gracilicornis*, might have started after female morphology evolved the extreme concealment of segment 8 within segment 7. Particularly suggestive are observations by Sattler [20] of mounting *A. najas* males hitting female antennae with alternating movements of the forward stretched mid-legs. This generally resembles leg movements by *G. gracilicornis*, with a crucial difference – *A. najas* males do not hit the water surface. Sattler [20] also observed that females protrude their genital segments in response to the male's stimulation. Hence, although not experimentally proven, it is possible that *A. najas* leg movements may have a similar function to the post-mounting ripple signals of *G. gracilicornis* (attachment signals). Although the degree of genitalia concealment in *A. najas* is less pronounced than in *G. gracilicornis*, it is considerable (S8/S6 = 0.47) in comparison to *A. remigis* (S8/S6 = 0.65), with coercive intromission in the absence of observed protrusion of S8 [2]. Hence, it is possible that once concealment of female genitalia reaches a point where the vulvar opening is at least partially shielded, as in *A. najas*, selection favoring post-mounting, pre-copulatory courtship behavior by males begins to operate.

However, in contrast to Sattler's observations [20], Arnqvist's thorough review [3] of all available information on mating behaviors of water striders resulted in classifying *A. najas* as a species practicing direct coercion, presumably in a manner similar to *A. remigis* [2], [24]. But, taking into consideration that only a few accounts (reviewed by [3]) contain good details of the pre-intromission interactions between the sexes, and that males of a species (*A. najas*) with a relatively low value (0.47) of S8/S6 appear to induce females to retract their gonocoxae and lower their ovipositor to facilitate intromission [20], we cautiously propose that intermediate degrees of genitalia concealment between those of *A. remigis* and *A. najas*, (0.65 to 0.47) might already provide females with some degree of success in opposing coercive intromissions. If this is true, then a coevolutionary arms race between the degree of female genitalia concealment and male genitalia morphology combined with signaling behavior is expected, in addition to the already studied evolution of female abdominal spines and other morphological traits [16], [17]. This hypothesis is currently being tested.

Why do *G. gracilicornis* females, with males already mounted on their backs, delay their response, and why do they protrude their genitalia only after receiving male ripple signals? We briefly describe here three mutually non-exclusive hypotheses based on the literature, and we treat this issue in detail in another paper [25]. First, the “protection from harassment” hypothesis proposes that females may forage more efficiently with mounted males producing signals, if the signals repel harassment from other males (e.g. [21], [26]). Because signaling stops after intromission is achieved, we believe that this hypothesis is not applicable here. Second, *G. gracilicornis* females may delay intromission in order to carefully assess the male's “quality,” and therefore may display more resistance to some males (physically and by delaying genitalia protrusion), and less resistant to others of better quality (Mate assessment hypothesis). Signal properties, including the amplitude or frequency of ripple signals, may carry information on the genetic qualities of males that may benefit a female's reproductive fitness. Third it has been established that frequent copulations are against the female's evolutionary interests due to the costs of mating [3], [27]–[29], and females attempt to throw the males off. Intromission gives the male an additional point of firm attachment to the female body, thereby helps the male in opposing the female's attempts to throw the male off. Hence, delaying intromission should make it easier for a female to throw off the mounted male, and avoid the costs of mating. Therefore, the delay of intromission may be viewed as one of the female's resistance strategies (Resistance hypothesis).

However, such resistance strategies are applicable when predation risk is low. Delaying intromission and demanding extra ripple signaling, as well as attempting to throw the male off before the intromission starts, may increase female vulnerability to predators, especially if predators are abundant. We proposed that this hypothetical sensitivity of female resistance strategies to the presence of predators may be contributing to the evolution of male signaling [25]. We hypothesize that, the males use signals to coerce the female by calling attention of predators to the mating. This hypothesis illustrates the role of predation risk in evolution of male signaling as a behavioral counter-adaptation to female's “victory” (evolution of genitalia concealment) in the evolutionary conflict between sexes over the initiation of mating. In the subsequent experiments we showed that male courtship signals induces females to protrude their genitalia in order to decrease the risk of attracting predators that cue on the ripple signals (CS Han and PG Jablonski, in preparation) and disproportionately increase the predation risk for females in comparison to males [27], [30], [31].

Regardless of the nature of the selection mechanisms shaping these signals, and regardless of their detailed evolutionary history, the analysis presented here strongly suggests that the evolution of post-mounting “intimate” signaling by coercive males has been triggered, in this species, by the ability of females to chose the timing of intromission - an outcome of genitalia concealment through morphological evolution of pre-genital segment 7. Previous research often demonstrated how antagonistic selection, triggered by evolutionary conflict between the sexes, leads to coevolution at the level of physiology or morphology [8], [12], [16], [17], [32]–[35]. Our results illustrate how the apparent “victory” of one sex in the antagonistic coevolution of morphological adaptations for intromission control (male and female genitalia) triggers the evolution of a behavioral trait in another sex (post-mounting courtship signals).

## Materials and Methods

### Study species

*Gerris gracilicornis* is widely distributed in East Asia, including Korea, Japan, and China. It inhabits temporary, stationary pools beside mountain streams, and mates in late March to early June. Eastern Palearctic *G. gracilicornis* group belongs to the *Macrogerris* subgenus which is located in the most basal clade of *Gerris* [36].

### Morphology of external female genitalia

We selected pairs that have remained in copula for at least five minutes and put them in liquid nitrogen or anaesthetized them in a cotton-filled transparent plastic box containing several drops of chloroform. Then, we immediately placed the pairs in a 70% ethanol solution to halt further deflation and retraction. After about 2 hours, we fixed the specimens with modified Karnovsky's fixative composed of 2% paraformaldehyde and 2% glutaraldehyde in a 0.05 M sodium cacodylate buffer (pH 7.2) for 4∼12 hours. After separating the abdominal tip from the rest of the body, the specimens were post-fixed with 1% osmium tetroxide in a 0.05 M sodium cacodylate buffer (pH 7.2) for 2 hours, after being washed three times with a 0.05 M sodium cacodylate buffer (pH 7.2). After a brief washing with distilled water, the fixed specimens were then dehydrated in an ethanol series, dried twice with isoaoamyl acetate for 15 min, critical-point dried, mounted on metal stubs, and coated with 10 nm of gold in a sputter-coater. We photographed them with a JEOL JSM-5410LV scanning electron microscope and a Carl Zeiss SUPRA 55VP field-emission scanning electron microscope. Mating pairs were also photographed using a digital camera (Sony A100) or digital camcorder (Sony SR-1) in order to analyze the inter-digitation of male and female reproductive organs.

### Observations of mating interactions and male signaling

The experimental individuals were collected at Gwanak Mountain near Seoul National University, between April 14, and June 8, 2008. After collecting, we separated them according to gender, and placed them in two rectangular plastic containers (40×50 cm). They were fed ad libitum with surplus frozen crickets (*Verlarifictorus asperses*) every two days. Pieces of floating Styrofoam were used as rest sites for the water striders. All animals were individually marked on the thorax with enamel paint. Twenty five pairs were closely observed in the laboratory to obtain detailed behavioral descriptions. For each observation session, a male and a female, which were kept in the lab for two to seven days without mating, were put in a transparent plastic container (15×15 cm). We closely monitored their interactions until complete intromission was achieved. We measured the timing of both signaling and intromission, and described the detailed behavioral context of the signaling. Additionally, four mating pairs were videotaped for later analysis to obtain detailed quantitative behavioral variables (Figure 4): 1) the number of pulses per bout, 2) the interval between pulses, and 3) the interval between bouts.

After we discovered that males produce pre-copulatory signals on the water's surface we set out to record these vibrations. The method of recording ripple signals relied upon the basic principle of Wilcox & Kashinsky [37]'s sensor. The recorder consisted of a small Styrofoam ball attached to the end of a classical voltmeter stylus, which was connected to an amplifier. The oscillatory movements of the ball on the water's surface were converted to electric signals by the recorder. Through the voltage amplifier, the electronic signals were recorded and saved in Waveform audio format. Noise in the recorded signals was reduced using Adobe Audition (© Adobe) which constructed a filter, automatically, based upon the vibrations present on the water's surface both before and after the signals were recorded. Spectrograms of recorded signals were produced using Spectrogram (© Visualization Software). For detailed analysis of signal production, the signaling behavior of four different males was analyzed from side-view recorded videos (30 frames per second) using MScope Player 2.21 (© Redlake).

### Experiment – function of male signals

*G. gracilicornis* males mounted on the backs of females often produce ripple signals by hitting the water's surface with their stretched mid-legs. To determine the effect of these signals on female behavior, a metal w-shaped bar (w-bar) was used to block the production of water surface waves by males. The bar did not interfere with female movements. The convex part in the middle of the w-bar was attached to the top of the female's thorax (Figure S1). The male's signals were blocked by the two concave parts located on both sides of the middle convex part. The presence of the w-bar substantially decreased the number of signals produced by males since males could not reach the water's surface. Only occasionally, when the mounted female lowered her body close to the water's surface (e.g. to drink), could males manage to reach the water's surface and produce signals.

To control for the effect of increased weight due to the presence of the w-bar, we used a control treatment: a straight bar of the same weight was attached to the female's back, parallel to the body axis. We measured the female's mating behavior using three treatments: control (natural) situation (*C*), the experimental “*w-bar*” attached to a female (*BF*), and a straight bar attached along the female's body (*nBF; non-effective bar*). Using 14 males, we confirmed that male behaviors after mounting were not affected by the *w-bar*: there was no difference in the frequency of the male's mid-leg movements (used for signal production) between normal matings and matings with w-bar equipped females (paired *t-*test, *t(13)* = 0.35, *p = *0.73). Hence, the main difference between treatments consisted of the presence or absence of ripple signals produced by males on the surface of water.

After collection, the experimental animals were individually marked on the thorax with enamel paint and separated, according to sex, into two rectangular plastic containers (40×50 cm). They were fed ad libitum with surplus frozen crickets (*Verlarifictorus asperses*) every two days. The experimental basin (15×30 cm) was divided into two compartments by an opaque partition. In each test, a male and a female were put in each compartment for an adjustment period. After three minutes, the partition was removed, and the two individuals allowed to mate. If the individuals initiated mating, we measured the duration of mating interaction (courtship duration in Figure 3D) from the moment a male grasped any part of the female's body (usually a leg) until successful intromission (when a male mounted the female and intromission was observed by an observer who kept close track of all behaviors of each tested pair).

Nine females were tested with a treatment order of “*C, BF, nBF*,” and thirteen females were tested in the order of “*C, nBF, BF*.” Treatment *C* always came first, since it was impossible to recreate the natural situation after applying glue in the *BF* and *nBF* treatments. The major test of the hypothesis lies in the comparison between *nBF* and *BF* treatments, since these treatments differ only in the presence, or absence, of ripple signals. Although two females died during “*C, BF, nBF*” treatment and one female died during “*C, nBF, BF*” treatment, we included them in statistical analyses of pair-wise comparisons between each of the two treatments. To reduce variation due to male weight, we used the extreme upper 30% of males according to a natural size distribution, with a body length between 12.6 and 13.4 mm. Males were randomly selected for the tests. We used the Wilcoxon matched pairs test to compare the BF treatment to the control treatment (C), the nBF treatment to the C treatment, and finally, the BF to the nBF treatment. Sequential Bonferroni correction of significance level was calculated for these three comparisons.

### Comparative analysis

The aim of the comparative analysis was to reconstruct the evolutionary history of the degree of exposure of female genitalia in Gerridae (segment 8), and to determine whether the degree of exposure is associated with the behavioral indications of the direct coercive mating system. Lower exposure, and hence, higher concealment, is viewed as an indicator of female control over the initiation of intromission.

Given that the second to sixth abdominal segments similarly contribute to abdomen length (they are “subequal” in Gerridae; [5]), and that abdomen length correlates with total body length (*A. remigis*, [38]), we believe that segment 6 can be used as a reference, by which the relative length of segment 8 can be measured for inter-specific comparisons. We chose S8/S6 ratio (segment 8 (gonocoxae)/segment 6; Figure 1) as an approximate index of the actual degree of exposure of female genitalia (S8/total S8; where total S8 also includes the part shielded in S7). Using fresh specimens of the three species available at our study site (*G. latiabdominis, G. gracilicornis*, and *A. paludum*) we confirmed that the S8/S6 ratio is an approximate indicator of the degree of protrusion of S8 (Figure S2). Lower values for the S8/S6 ratio imply that the gonocoxae are relatively well concealed within segment 7. Digital photos of the genitalia of dried female specimens from 17 species of the genus *Gerris, Aquarius*, and *Limnoporus* (Table S2) were analyzed with the program Image J. We confirmed that the S8/S6 ratio from dried and fresh females did not differ (*G. latiabdominis*, Paired *t-*test, *t(8)* = 0.84, *p* = 0.43; *G. gracilicornis*, *t(6)* = 1.05, *p* = 0.34). Digital images of individuals were obtained using a digital camera (Sony A100) or a digital camera attached to a dissecting microscope. Each segment length was measured in the ventral position along the midline of the body.

Ancestral states were reconstructed using a phylogenetic tree derived from Damgaard and Cognato [6]. The tree was a strict consensus of eight parsimonious trees, and branch lengths were determined by Damgaard and Cognato [6] based upon changes within 2,356 molecular and morphological characters.

We used COMPARE version 4.6 [39] for ancestor reconstructions of the continuous trait, S8/S6 ratio. Ancestral states were calculated using the phylogenetic generalized least squares (PGLS) ancestor method [40], [41]. A Brownian motion model of evolution was assumed. Within-species variation was set to zero, but branch lengths were proportional to substitutional changes derived from Damgaard and Cognato [6]. Statistically significant character changes were calculated using standard errors according to Rohlf [42], included in the COMPARE software.

Based on available information, we classified mating systems into three types: direct coercion (DC; 13 species), direct coercion mixed with persuasive mating with pre-mounting courtship signaling (DC/P1; 3 species), and persuasive mating with post-mounting courtship signaling (P2; *G. gracilicornis*, this study). According to the published evidence, most water striders employ the “Direct coercive mating (DC)” strategy exemplified by detailed observations of *A. remigis* [2]; a male water strider mounts a female and inserts his genitalia forcefully into the female genital tract. Within this general category, some species (*A. elongatus* or *Limnoporus sp.*) have been described to alternatively use courtship ripple signals to advertise their presence to females, who approach signaling males [3], [43]–[49] and enter into copulation. This mating behavior fits both classical courtship behavior and “persuasive mating” behavior [1], due to the fact that females control mating initiation and males cannot copulate without female cooperation. We classified it as “Persuasive mating type 1 mixed with direct coercion (DC/P1).” Given the evidence presented here, *G. gracilicornis*, is the only species of Gerridae in which males, after forcefully mounting a female, produce intimate courtship signals to induce (“persuade” sensu [1]) the protrusion of female genitalia (a unique “post-mounting pre-copulatory courtship” of *G. gracilicornis*). We labeled this mating behavior “Persuasive mating type 2 (P2).”

For the reconstruction of discrete characters (three mating behavior types: DC, DC/P1, P2), we conducted parsimony reconstruction using Mesquite, version 2.5 [50]. The three mating system types are considered to be non-ordered. We used accounts on water strider mating behaviors summarized in Arnqvist [3] and our own observations. A Brownian motion model of evolution was assumed, and the within-species variation was set to zero and all branch lengths were assumed to equal 1.

## Supporting Information

Figure S1 Female Gerris gracilicornis with w-shaped bar (w-bar). W-bar was attached to female thorax using super-glue. The bar blocks the production of males' ripple signals.
(1.62 MB TIF)

Figure S2 S8/S6 ratio (see Figure 1 for definition of S8 and S6) as an index of the proportion of exposure of S8. Comparison between S8/S6 ratio and the directly measured proportion of the total length of S8 that is exposed (length of exposed S8/total length of S8 measured after removing it from the shield of S7) for three species collected at the field site: Gerris gracilicornis (GG), Gerris latiabdominis (GL), Aquarius paludum (AP). Means and standard deviations are shown.
(0.40 MB TIF)

Table S1 Characteristics of the three types of ripple waves produced by mid-leg movements of male G. gracilicornis and their comparison with other ripple signals known for Gerridae. #/Bout - the number of pulses per bout; INTERVAL P - interval (sec) between pulses in one bout; INTERVAL B - interval (sec) between bouts. Refer to Figure 3 and the main text for further descriptions of the variables. The individual 2 produced only one bout of the mounting signals. Therefore, the interval between bouts could not be measured. Statistics for Table S1 The three types of signals (Table S1) differed among each other with respect to some aspects of each of the three variables: 1) the number of pulses per bout, 2) the interval between pulses, and 3) the interval between bouts. We used two-way ANOVA to test the effects of signal type (3 types: grasping signals, mounting signals, and attachment signals; see Results) and individual identity (4 individuals) on variables (2) and (3). Further post-hoc comparisons were conducted using unequal N Tukey honest significant difference (HSD) tests. We also tested for differences in the coefficient of variation between signal-types [53]. We used General Linearized Modeling with Poisson distribution and identity link functions to test the effects of signal type (3 types: grasping signals, mounting signals, and attachment signals; see Results) and individual identity (4 individuals) on the number of pulses per bout. Although, the three signal types did not differ in the number of pulses per bout (W2,37 = 0.01, p = 0.99; Wald Statistitcs in GLZ with Poisson distribution and identity link function: effect of individual identity: W3,37 = 5.76, p = 0.12; interaction “individual x signal types”: W6,37 = 2.63, p = 0.85), the number of pulses per bout was more variable in the case of attachment signals than in that of grasping signals (Z = −2.22, p = 0.03; test for differences between coefficients of variation, [53]). The interval between pulses in a bout differed (log-transformed data: F2,130 = 18.18, p<0.0001) among the signal types (log-transformed data: interaction “individual x signal types”: F6,130 = 1.5, p = 0.182; effect of individual identity: F3,130 = 2.17, p = 0.10): the interval was shorter in the attachment than in the grasping (Unequal N Tukey HSD test: P<0.0001) or mounting (p<0.0001) signals. Although the interval between bouts showed no difference among signal types (F2,22 = 0.23, p = 0.8), it was less variable in the attachment than in the grasping (Z = −3.12, p = 0.002) or mounting signals (Z = 3.10, p = 0.002). Comparison with literature on ripple signals in water striders The post-mounting, pre-copulatory courtship signals of G. gracilicornis males appear to be quite unique among Gerridae with a direct coercive mating system. They are different from the signals of males used during copulation and/or guarding (i.e. copulatory and post-copulatory signals) in G. lacustris [22], A. remigis [21], [26] and G. lateralis [23], or for defense of resources in A. remigis [54]. Given the published evidence, these species are known for their direct coercive mating system, and the morphology of segment 8 indicates that, unlike in G. gracilicornis (S8/S6 = 0.28), female genitalia remain largely exposed and susceptible to forceful intromission by males (S8/S6 in most species is larger than 0.5). The signals of these species were hypothesized to ward off single males from the mating pair. They may also function as post-copulatory courtship, common among many insects [55]. Post-mounting courtship signals of G. gracilicornis also clearly differ in their context, as well as in frequency, from the courtship signals of A. elongatus and Limnoporus sp. with pre-mounting courtship signals, (DC/P1 in Figure 4), where males attract females to oviposition sites using pre-mounting ripple signals [3], [43]–[49].
(0.04 MB PDF)

Table S2 S8/S6 and S7/S6 ratios with sample sizes for each of the 17 species presented in Figure 4 (see Figure 1 for definitions of S6, S7, and S8).
(0.01 MB PDF)

Video S1 Three types of courtship signals produced by G. gracilicornis males. Grasping signals, mounting signals, and attachment signals.
(6.56 MB MOV)

Video S2 A mounted male producing attachment signals by pressing the female abdomen tip without inserting his genitalia.
(4.79 MB MOV)

Video S3 A video showing how a female exposes the genitalia after the attachment signals.
(9.71 MB MOV)
