# Supplementary figures and images for: Female Genitalia Concealment Promotes Intimate Male Courtship in a Water Strider
Source: PLoS One. 2009 Jun 10;4(6):e5793. doi: 10.1371/journal.pone.0005793 (PMC2686155; doi:10.1371/journal.pone.0005793)

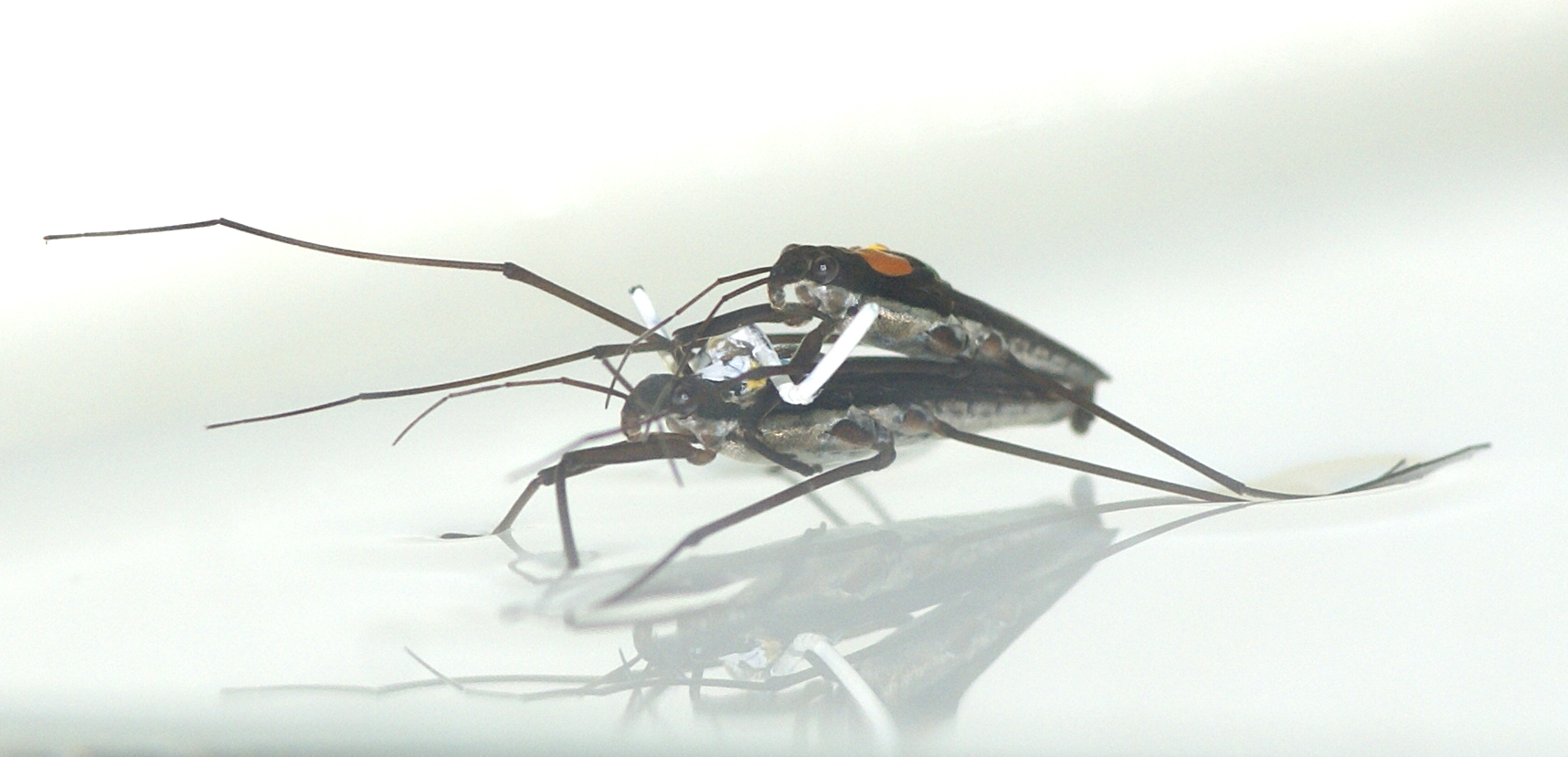

Supplement: Figure S1 — Female Gerris gracilicornis with w-shaped bar (w-bar). W-bar was attached to female thorax using super-glue. The bar blocks the production of males' ripple signals. (1.62 MB TIF) [file pone.0005793.s001.tif]

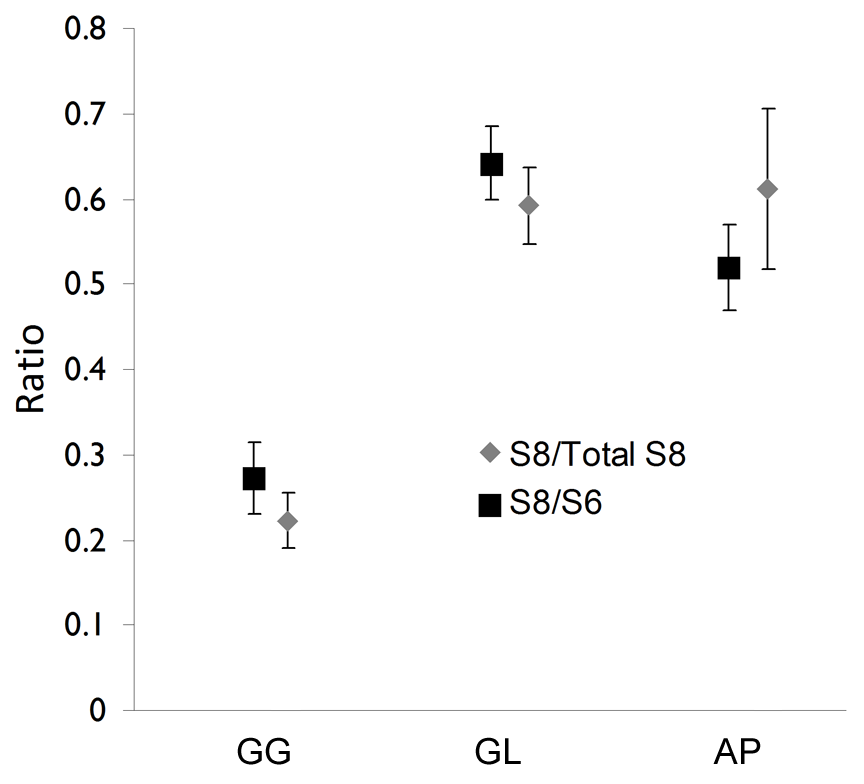

Supplement: Figure S2 — S8/S6 ratio (see Figure 1 for definition of S8 and S6) as an index of the proportion of exposure of S8. Comparison between S8/S6 ratio and the directly measured proportion of the total length of S8 that is exposed (length of exposed S8/total length of S8 measured after removing it from the shield of S7) for three species collected at the field site: Gerris gracilicornis (GG), Gerris latiabdominis (GL), Aquarius paludum (AP). Means and standard deviations are shown. (0.40 MB TIF) [file pone.0005793.s002.tif]
