# Supplementary material for: Female Genitalia Concealment Promotes Intimate Male Courtship in a Water Strider
Source: PLoS One. 2009 Jun 10;4(6):e5793. doi: 10.1371/journal.pone.0005793 (PMC2686155; doi:10.1371/journal.pone.0005793)
Supplement: Table S1 — Characteristics of the three types of ripple waves produced by mid-leg movements of male G. gracilicornis and their comparison with other ripple signals known for Gerridae. #/Bout - the number of pulses per bout; INTERVAL P - interval (sec) between pulses in one bout; INTERVAL B - interval (sec) between bouts. Refer to Figure 3 and the main text for further descriptions of the variables. The individual 2 produced only one bout of the mounting signals. Therefore, the interval between bouts could not be measured. Statistics for Table S1 The three types of signals (Table S1) differed among each other with respect to some aspects of each of the three variables: 1) the number of pulses per bout, 2) the interval between pulses, and 3) the interval between bouts. We used two-way ANOVA to test the effects of signal type (3 types: grasping signals, mounting signals, and attachment signals; see Results) and individual identity (4 individuals) on variables (2) and (3). Further post-hoc comparisons were conducted using unequal N Tukey honest significant difference (HSD) tests. We also tested for differences in the coefficient of variation between signal-types [53]. We used General Linearized Modeling with Poisson distribution and identity link functions to test the effects of signal type (3 types: grasping signals, mounting signals, and attachment signals; see Results) and individual identity (4 individuals) on the number of pulses per bout. Although, the three signal types did not differ in the number of pulses per bout (W2,37 = 0.01, p = 0.99; Wald Statistitcs in GLZ with Poisson distribution and identity link function: effect of individual identity: W3,37 = 5.76, p = 0.12; interaction “individual x signal types”: W6,37 = 2.63, p = 0.85), the number of pulses per bout was more variable in the case of attachment signals than in that of grasping signals (Z = −2.22, p = 0.03; test for differences between coefficients of variation, [53]). The interval between pulses in a bout dif [file pone.0005793.s003.pdf]

Table S1. Characteristics of the three types of ripple waves produced by mid-leg movements of male *G. gracilicornis* and their comparison with other ripple signals known for Gerridae.

	Grasping Signals			Mounting Signals			Attachment Signals		
INDIVIDUAL	#/BOUT	INTERVAL P	INTERVAL B	#/BOUT	INTERVAL P	INTERVAL B	#/BOUT	INTERVAL P	INTERVAL B
1	5.2 ± 3.6	0.58 ± 0.40	1.78 ± 0.36	4.8 ± 2.1	0.55 ± 0.17	2.64 ± 2.57	4.0 ± 1.0	0.30 ± 0.08	3.22 ± 0.40
2	3.0 ± 2.3	0.58 ± 0.36	1.07 ± 0.30	4.0 ± 0.0	0.34 ± 0.16		4.7 ± 1.5	0.41 ± 0.16	2.24 ± 1.27
3	2.8 ± 1.0	0.48 ± 0.19	2.44 ± 1.71	3.0 ± 1.4	0.47 ± 0.27	1.27 ± 0.00	2.8 ± 0.8	0.31 ± 0.19	2.39 ± 0.28
4	4.8 ± 1.9	0.51 ± 0.32	4.61 ± 1.60	4.0 ± 2.6	0.44 ± 0.24	3.65 ± 1.67	4.0 ± 1.2	0.26 ± 0.07	3.09 ± 0.34

#/Bout - the number of pulses per bout; INTERVAL P - interval (sec) between pulses in one bout; INTERVAL B - interval (sec) between bouts. Refer to Figure 4 and the main text for further descriptions of the variables. The individual 2 produced only one bout of the mounting signals. Therefore, the interval between bouts could not be measured.

Statistics for Table S1

The three types of signals (Table S1) differed among each other with respect to some aspects of each of the three variables: 1) the number of pulses per bout, 2) the interval between pulses, and 3) the interval between bouts. We used two-way ANOVA to test the effects of signal type (3 types: grasping signals, mounting signals, and attachment signals; see Results) and individual identity (4 individuals) on variables (2) and (3). Further post-hoc comparisons were conducted using unequal N Tukey honest significant difference (HSD) tests. We also tested for differences in the coefficient of variation between signal-types [46]. We used General Linearized Modeling with Poisson distribution and identity link functions to test the effects of signal type (3 types: grasping signals, mounting signals, and attachment signals; see Results) and individual identity (4 individuals) on the number of pulses per bout. Although, the three signal types did not differ in the number of pulses per bout ($W_{2,37}$=0.01, p=0.99; Wald Statistitcs in GLZ with Poisson distribution and identity link function: effect of individual identity: $W_{3,37}$=5.76, p=0.12; interaction "individual x signal type": $W_{6,37}$=2.63, p=0.85), the number of pulses per bout was more variable in the case of attachment signals than in that of grasping signals (Z=-2.22, p=0.03; test for differences between coefficients of variation, [46]). The interval between pulses in a bout differed (log-transformed data: $F_{2,130}$=18.18, p<0.0001) among the signal types (log-transformed data: interaction between individuals and signal types: $F_{6,130}$ =1.5, p=0.182; effect of individual identity: $F_{3,130}$=2.17, p=0.10): the interval was shorter in the attachment than in the grasping (Unequal N Tukey HSD test: P<0.0001) or mounting (p<0.0001) signals. Although the interval between bouts showed no difference

among signal types ($F_{2,22}$=0.23, p=0.8), it was less variable in the attachment than in the grasping (Z=-3.12, p=0.002) or mounting signals (Z=3.10, p=0.002).

Comparison with literature on ripple signals in water striders

The post-mounting, pre-copulatory courtship signals of *G. gracilicornis* males appear to be quite unique among Gerridae with a direct coercive mating system. They are different from the signals of males used during copulation and/or guarding (i.e. copulatory and post-copulatory signals) in *G. lacustris* [47], *A. remigis* [20,21] and *G. lateralis* [48], or for defense of resources in *A. remigis* [49]. Given the published evidence, these species are known for their direct coercive mating system, and the morphology of segment 8 indicates that, unlike in *G. gracilicornis* (S8/S6 = 0.28), female genitalia remain largely exposed and susceptible to forceful intromission by males (S8/S6 in most species is larger than 0.5). The signals of these species were hypothesized to ward off single males from the mating pair. They may also function as post-copulatory courtship, common among many insects [50].

Post-mounting courtship signals of *G. gracilicornis* also clearly differ in their context, as well as in frequency, from the courtship signals of *A. elongatus* and *Limnoporus sp.* with pre-mounting courtship signals, (DC/P1 in Figure 6), where males attract females to oviposition sites using pre-mounting ripple signals [3,36-42].
