# Supplementary material for: Female Genitalia Concealment Promotes Intimate Male Courtship in a Water Strider
Source: PLoS One. 2009 Jun 10;4(6):e5793. doi: 10.1371/journal.pone.0005793 (PMC2686155; doi:10.1371/journal.pone.0005793)
Supplement: Table S2 — S8/S6 and S7/S6 ratios with sample sizes for each of the 17 species presented in Figure 4 (see Figure 1 for definitions of S6, S7, and S8). (0.01 MB PDF) [file pone.0005793.s004.pdf]

Table S2. S8/S6 and S7/S6 ratios with sample sizes for each of the 17 species presented in Figure 6 (see Figure 1 for definitions of S6, S7, and S8).

	S8/S6 (mean±SD)	S7/S6 (mean±SD)	Number of samples	Sampling Site
G. lacustris	*0.67 ± 0.02*	*1.01 ± 0.06*	3	Palaearctic, Cornell University Insect Collection
G. comatus	*0.70 ± 0.04*	*1.04 ± 0.05*	4	Nearctic, Cornell University Insect Collection
G. marginatus	*0.68 ± 0.06*	*0.98 ± 0.07*	6	Nearctic, Cornell University Insect Collection
G. inseperatus	*0.64 ± 0.06*	*1.01 ± 0.08*	5	Nearctic, Cornell University Insect Collection
G. latiabdominis	*0.63 ± 0.04*	*0.97 ± 0.06*	34	Kimpo Ricefields, Seoul, Korea
G. buenoi	*0.76 ± 0.08*	*1.25 ± 0.10*	7	Nearctic, Cornell University Insect Collection
G. pingreensis	*1.07 ± 0.04*	*1.28 ± 0.11*	3	Nearctic, Cornell University Insect Collection
G. incognitus	*0.98 ± 0.21*	*1.09 ± 0.10*	6	Nearctic, Cornell University Insect Collection
G. gracilicornis	*0.28 ± 0.03*	*1.30 ± 0.07*	155	Gwanak Mountain, Seoul, Korea
A. remigis	*0.65 ± 0.12*	*1.39 ± 0.07*	8	Nearctic, Cornell University Insect Collection
A. paludum	*0.52 ± 0.10*	*0.99 ± 0.05*	21	Gwanak Mountain, Seoul, Korea
A. conformis	*0.64 ± 0.07*	*1.10 ± 0.04*	3	Nearctic, Cornell University Insect Collection
A. nebularis	*0.61 ± 0.06*	*1.27 ± 0.06*	12	Nearctic, Cornell University Insect Collection
A. elongatus	*0.45 ± 0.03*	*0.76 ± 0.01*	3	Jiri Mountain, Jeollanam-do, Korea
A. najas	*0.46 ± 0.03*	*0.93 ± 0.03*	3	Kurowka river near Pulawy, Lublin district, Poland
L. dissortis	*0.68 ± 0.06*	*0.89 ± 0.03*	5	Nearctic, Cornell University Insect Collection
L. notabilis	*0.60 ± 0.06*	*0.86 ± 0.16*	6	Nearctic, Cornell University Insect Collection
